# Rodent Thyroid, Liver, and Fetal Testis Toxicity of the Monoester Metabolite of Bis-(2-ethylhexyl) Tetrabromophthalate (TBPH), a Novel Brominated Flame Retardant Present in Indoor Dust

**DOI:** 10.1289/ehp.1204932

**Published:** 2012-09-26

**Authors:** Cecilia Springer, Edward Dere, Susan J. Hall, Elizabeth V. McDonnell, Simon C. Roberts, Craig M. Butt, Heather M. Stapleton, Deborah J. Watkins, Michael D. McClean, Thomas F. Webster, Jennifer J. Schlezinger, Kim Boekelheide

**Affiliations:** 1 Department of Pathology and Laboratory Medicine, Brown University, Providence, Rhode Island, USA; 2 Division of Urology, Rhode Island Hospital, Providence, Rhode Island, USA; 3 Nicholas School of the Environment, Duke University, Durham, North Carolina, USA; 4 Department of Environmental Health, Boston University School of Public Health, Boston, Massachusetts, USA; 5 Center for Environmental Health and Technology, Brown University, Providence, Rhode Island, USA

**Keywords:** brominated, exposure, flame retardant, hepatotoxicity, hypothyroidism, metabolism, phthalate, PPAR, toxicity

## Abstract

Background: Bis-(2-ethylhexyl) tetrabromophthalate (TBPH) is widely used as a replacement for polybrominated diphenyl ethers (PBDEs) in commercial flame retardant mixtures such as Firemaster 550. It is also used in a commercial mixture called DP 45. Mono-(2-ethyhexyl) tetrabromophthalate (TBMEHP) is a potentially toxic metabolite.

Objectives: We used *in vitro* and rodent *in vivo* models to evaluate human exposure and the potential metabolism and toxicity of TBPH.

Methods: Dust collected from homes, offices, and cars was measured for TBPH by gas chromatography followed by mass spectrometry. Pregnant rats were gavaged with TBMEHP (200 or 500 mg/kg) or corn oil on gestational days 18 and 19, and dams and fetuses were evaluated histologically for toxicity. We also assessed TBMEHP for deiodinase inhibition using rat liver microsomes and for peroxisome proliferator-activated receptor (PPAR) α and γ activation using murine FAO cells and NIH 3T3 L1 cells.

Results: TBPH concentrations in dust from office buildings (median, 410 ng/g) were higher than in main living areas in homes (median, 150 ng/g). TBPH was metabolized by purified porcine esterases to TBMEHP. Two days of TBMEHP exposure in the rat produced maternal hypothyroidism with markedly decreased serum T3 (3,3´,5-triiodo-l-thyronine), maternal hepatotoxicity, and increased multinucleated germ cells (MNGs) in fetal testes without antiandrogenic effects. *In vitro*, TBMEHP inhibited deiodinase activity, induced adipocyte differentiation in NIH 3T3 L1 cells, and activated PPARα- and PPARγ-mediated gene transcription in NIH 3T3 L1 cells and FAO cells, respectively.

Conclusions: TBPH *a*) is present in dust from indoor environments (implying human exposure) and *b*) can be metabolized by porcine esterases to TBMEHP, which *c*) elicited maternal thyrotoxic and hepatotoxic effects and *d*) induced MNGs in the fetal testes in a rat model. In mouse NIH 3T3 L1 preadipocyte cells, TBMEHP inhibited rat hepatic microsome deiodinase activity and was an agonist for PPARs in murine FAO and NIH 3T3 L1 cells.

Brominated flame retardants (BFRs) have been routinely added to a wide range of consumer products to reduce their inherent flammability and thus lower potential fire-related injuries and property damage. However, there is growing concern regarding their toxic effects (Birnbaum and Staskal 2004) because of their environmental persistence and measurably elevated levels in humans (Meneses et al. 1999; Sjödin et al. 1999).

Until recently, polybrominated diphenyl ethers (PBDEs) were the most commonly used class of flame retardants (de Wit 2002). PBDEs have high structural similarity to the endogenous thyroid hormones 3,5,3´,5´-tetraiodo-l-thyronine [thyroxine, (T4)] and 3,3´,5-triiodo-l-thyronine [triiodothyronine (T3)] (Schriks et al. 2007) resulting in the potential to disrupt thyroid hormone homeostasis (Butt et al. 2011; Darnerud et al. 2001). Rodents acutely exposed to PBDE (Hallgren and Darnerud 2002; Zhou et al. 2001) and environmentally exposed human populations (Bloom et al. 2008; Hagmar et al. 2001; Julander et al. 2005; Turyk et al. 2008; Yuan et al. 2008) have disrupted thyroid activity and function. Concerns over environmental persistence, neurotoxicity, and endocrine-disrupting effects have led to legislative restrictions on PBDE use (Costa et al. 2008). By 2010, the U.S. Environmental Protection Agency (EPA) instituted phase-out programs for all types of PBDE flame retardants (U.S. EPA 2009b).

With a continuing demand for flame retardants, various PBDE replacements have been introduced into commerce. Firemaster 550 (Chemtura Corp., Middlebury, CT) is a new flame retardant mixture; although its composition remains proprietary, bis-(2-ethylhexyl) tetrabromophthalate (TBPH) has been identified as a component (Stapleton et al. 2008). TBPH, an ingredient in several other Chemtura flame retardant mixtures, is used in PVC (polyvinyl chloride), neoprene, wire insulation, carpet backing, coated fabrics, and wall coverings (Covaci et al. 2011). TBPH is a high production volume chemical with 450–4,500 tons produced in 2006 (Covaci et al. 2011). Its widespread use is clearly evident in the detectable levels of TBPH found in household dust (Stapleton et al. 2008) and rapidly rising atmospheric levels in the North American Great Lakes region (Ma et al. 2011). Furthermore, TBPH has been discovered in the fatty tissue of higher-order species, including porpoises and dolphins in the South China Sea, indicating its ability to enter the environment (Lam et al. 2009).

The U.S. EPA’s High Production Volume Information System (U.S. EPA 2009a) is the sole source of information on the toxicity of TBPH. TBPH has a half-life of approximately 29 days in water (25°C, pH 7). Studies of acute toxicity in rats showed that a single dose of 5,000 mg/kg did not produce lethality, outwardly observable effects, or gross changes at necropsy after 15 days. Toxicity following chronic exposure was tested by food-borne exposure for 28 days in a rat model (U.S. EPA 2009a). The reported no observed adverse effect level was 2,000 ppm (223 mg/kg/day). High-dose exposure to 20,000 ppm TBPH (2,331 mg/kg/day) significantly decreased serum alanine amino transferase, calcium, and phosphorus levels and decreased body weight. *In vivo* studies in mice failed to identify genotoxic effects in the form of micronucleated erythrocytes in the bone marrow after either dermal exposure or intraperitoneal injection. *In vitro* studies using isolated human lymphocytes also did not exhibit any elicited chromosomal aberrations (U.S. EPA 2009a).

TBPH is a structural analog of di(2-ethylhexyl) phthalate (DEHP), a known peroxisome proliferator and male reproductive toxicant in rodents. DEHP is metabolized by esterases to mono(2-ethylhexyl) phthalate (MEHP), its toxicologically active monoester metabolite (Figure 1). DEHP induces hepatotoxicity in rodents, most likely as a result of MEHP-induced activation of peroxisome proliferator activated receptor α (PPARα) (Ward et al. 1998). The developing male reproductive system in rats is highly sensitive to the effects of these phthalates, which decrease fetal male testosterone levels (Parks et al. 2000). The active phthalates disrupt steroidogenesis in fetal rat Leydig cells, and this antiandrogenic effect impairs the normal development of the male reproductive tract (Jones et al. 1993; Lehmann et al. 2004; Liu et al. 2005; Shultz et al. 2001). The active phthalates also alter fetal testis seminiferous cords, an effect manifested by the induction of multinucleated germ cells (MNGs) (Boekelheide et al. 2009; Gaido et al. 2007).

**Figure 1:**
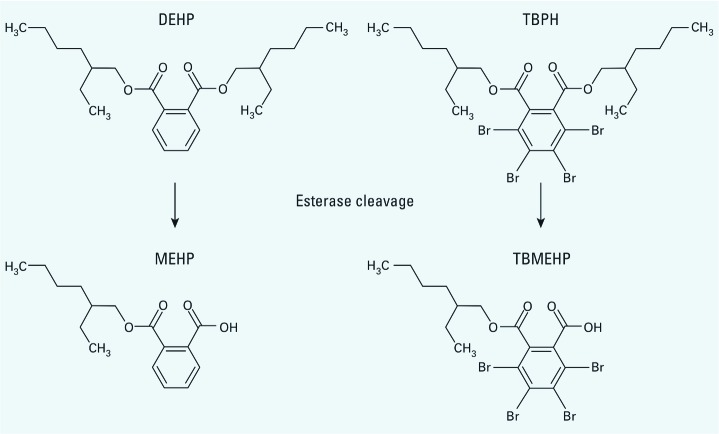
Molecular structures of DEHP and TBPH and cleavage by hydrolysis or esterases to their monoester metabolites, MEHP and TBMEHP.

Because of the similarity of TBPH to the known developmental reproductive toxicant DEHP, in the present study we focused on this component of Firemaster 550. The detectable presence of TBPH in the environment and its use in commonly encountered materials in homes and offices led us to assess human exposure levels. Because of the similarities in chemical structures of DEHP and its active monoester metabolite (MEHP) with TBPH and its potentially toxicologically active monoester metabolite, mono(2-ethyhexyl) tetrabromophthalate (TBMEHP; Figure 1), we focused on characterizing the toxic effects of TBMEHP. We examined the generation of TBMEHP from TBPH by *in vitro* incubation with porcine esterases. We then investigated TBMEHP *in vivo* using an *in utero* exposure paradigm in rats, and *in vitro* with mechanistic studies using murine FAO and NIH 3T3 L1 cells to evaluate its potential toxicity.

## Materials and Methods

*Chemicals*. TBPH and TBMEHP were synthesized by AsisChem (Watertown, MA). We purchased MEHP from TCI America (Portland, OR); rosiglitazone from Cayman Chemical (Ann Arbor, MI); and 2-(4-(2-(1-(cyclohexanebutyl)-3-cyclohexylureido)ethyl)phenylthio)-2-methylpropionic acid (GW7647), a prototypic PPARα agonist, was purchased from Sigma-Aldrich (St. Louis, MO).

*Exposure assessment*. We sampled the homes, offices, and cars of 31 participants in the Boston, Massachusetts, area during the winter of 2009. The institutional review board of the Boston University Medical Center approved the study protocol, and all participants gave their informed consent. Dust samples were collected by investigators using a cellulose extraction thimble (Whatman International, Piscataway, NJ) inserted between the crevice tool and vacuum tube extender of a Eureka Mighty-Mite vacuum cleaner (Allen et al. 2008). Each main living area of the home and each office was vacuumed for approximately 10 min, capturing dust from the surface area of the room. Cars (*n* = 20) were vacuumed for approximately 10 min, collecting dust from the entire surface of the front and back seats. The dashboard, floor, and other surfaces of the vehicles were not vacuumed. Dust samples were sieved to collect particles < 500 μm in size. The sieved samples were placed in clean amber glass jars and stored at –20°C until analysis. Sodium sulfate powder was used as a surrogate for dust in the collection of field blanks. Samples were analyzed for TBPH using gas chromatography–mass spectrometry (GC-MS) as previously described (Stapleton et al. 2008). An indoor dust standard reference material (SRM 2585; National Institute of Standards and Technology, Gaithersburg, MD), field blanks (*n* = 12) and laboratory blanks (sodium sulfate, *n* = 3) were all run alongside the dust samples for quality control purposes. TBPH was not detected in any field or laboratory blank. The limit of detection (LOD) was based on the instrumental detection limit using a signal to noise ratio of 3 (2.5 ng). Concentrations below the LOD were substituted with a value of one-half the LOD. The concentration of TBPH in SRM 2585 was 779 ± 108 ng/g, which is within the range reported by two other research laboratories (Sahlström et al. 2011; Van den Eede et al. 2012).

*TBPH metabolism*. *In vitro* metabolism experiments were performed using commercially available purified hepatic porcine esterase (Sigma-Aldrich) to assess the potential metabolism of TBPH and quantify the formation rate of TBMEHP. Purified human and rat esterases were not available. Enzymatic incubations were performed in 1 mL of 0.1 M potassium phosphate buffer (pH 7.4) for 2 hr at 37°C in the presence of 5.6 μM TBPH delivered in 1 μL of DMSO and 0.1 mg of porcine hepatic esterase. We selected the concentration of TBPH used to maximize detection of potential metabolites without greatly exceeding the aqueous solubility. Lower concentrations of TBPH were evaluated for metabolism under similar conditions but resulted in no detectable levels of TBMEHP. The concentration of esterases used was also maximized based on the stock solution available. Reactions were stopped by the addition of 1 mL of 1 M hydrochloric acid. Before extraction, 50 ng of tetrachloro-monohexyl phthalate (TCMHP; Sigma-Aldrich) was added to each sample as an internal quantitation standard. Solid-phase extraction was performed with Sampli-Q OPT cartridges (3 mL, 30 mg; Agilent Inc., Santa Clara, CA), which were first conditioned with 3 mL methanol (MeOH) and rinsed with 3 mL water. Samples were added to the cartridges, and the cartridges were rinsed with 3 mL water; the analytes were then eluted with 3 mL MeOH. Low rates of nonenzymatic formation of TBMEHP (< 2% of active samples) detected in control incubations without esterase were subtracted from values observed in active incubations. Analysis of TBMEHP was performed using liquid chromatography with tandem mass spectrometry (LC/MS/MS) with negative electrospray ionization. All samples were prepared in duplicate, and experiments were repeated on 2 separate days. *In vitro* TBPH metabolism has been described in detail by Roberts et al. (2012).

In vivo *rat experiments.* Timed-pregnant Fischer rats were purchased from Charles River Laboratories (Wilmington, MA). Rats were housed at 21 ± 1°C on a 12-hr light/dark cycle; food and water were given *ad libitum.* All animal procedures were performed with approval from Brown University’s Institutional Animal Care and Use Committee, and animals were treated humanely and with regard for alleviation of suffering. Rats were assigned by randomized weight to three treatment groups: corn oil/ethanol control (*n* = 10), 200 mg/kg TBMEHP (low dose; *n* = 9), and 500 mg/kg TBMEHP (high dose; *n* = 10). Rats were housed in random pairs within groups. TBMEHP, which is a water-insoluble solid at room temperature, was dissolved in a solution of corn oil and 5% ethanol. Dosing was by gavage (2 mL/kg).

Rat dams were dosed once per day on gestational days (GDs) 18 and 19. Six hours after the final dose, dams were euthanized by isoflurane overdose and cervical dislocation. Blood was collected by cardiac puncture, and serum was isolated by centrifugation and then frozen at –80°C until further use. Organs were harvested and weighed, then fixed in 10% neutral buffered formalin. Fetuses were euthanized by decapitation, and sex was identified by internal dissection. Within each litter, both testes of male pups were numbered upon isolation. The first testis was placed into formalin, the second and third testes were snap frozen in liquid N_2_, and the fourth testis was incubated in media for a 3-hr testosterone production assay. With the fifth testis, the sequence was repeated, ensuring an equal and randomized assignment of testes within a litter for various analyses.

Dam ovaries, adrenal glands, kidneys, and livers were isolated and weighed. The thyroid was harvested by excising the upper part of the trachea. The kidneys, livers, and thyroids were embedded in paraffin, sectioned (5 µm), and stained with hematoxylin and eosin (H&E). We scored livers for the presence of mitotic cells by counting the number of characteristic events (cells with chromosomal condensation and aggregation, and nuclear envelope breakdown) in five randomly selected high-powered fields per liver section. The average number of mitotic cells per high-powered field per dam was then converted to a square millimeter unit area and used to compare across groups. This morphological assessment was complemented by immunohistochemical staining using a rabbit monoclonal antibody to the Ki67 (catalog no. ab16667; Abcam, PLC, Cambridge, MA) proliferation marker on paraffin-embedded liver sections from the 29 dams. Ki67 is expressed during all phases of the cell cycle but does not quantify how many cells will undergo mitosis. Slides were scanned using an Aperio CS Scanscope (Aperio Technologies Inc., Vista, CA); 20 randomly selected fixed-size fields from each section were selected for quantification by overlaying the tissue with a numbered grid. Cells were counted as proliferative events regardless of stain intensity if the stained area was > 3 µm in length, if at least half of the stained area was within the fixed-size area, and if there was a clear central area of the stain that was more intensely stained than the rest. We used the area of the fixed-size fields to calculate Ki67-positive staining per square millimeter, and then averaged the measurements for each slide. The slide averages were pooled within treatment groups and used to compare across groups.

Liver apoptosis was quantified by terminal deoxynucleotidyl transferase dUTP nick end labeling (TUNEL) staining. Livers were embedded in paraffin; 5-µm tissue sections were stained using an ApopTag Peroxidase In Situ Apoptosis Detection Kit (catalog no. S7100; Chemicon International, Temecula, CA) according to the manufacturer’s protocol. The number of TUNEL-positive cells per high-powered field (400×) was determined in 20 randomly selected fields from the liver sections from each dam. The area of the high-powered field was used to calculate the number of TUNEL-positive cells per square millimeter. We then used the average number of positive cells per dam per square millimeter to compare across treatment groups.

Maternal serum was analyzed for liver enzymes (alkaline phosphatase, alanine aminotransferase, and aspartate aminotransferase), albumin, uric acid, total protein, glucose, triglycerides, carbon dioxide, creatinine, blood urea nitrogen, calcium, chloride, sodium, potassium, phosphorus, and cholesterol with a Beckman Coulter DxC analyzer (Beckman Coulter, Brea, CA). Thyroid hormones (T3 and T4) were measured using a Siemens ADVIA Centaur XP assay system (Siemens AG, Munich, Germany).

We examined the effects of *in utero* exposure on the fetal testes. Formalin-fixed fetal testes were embedded in glycol methacrylate (Heraeus Kulzer, LLC, Wehrheim, Germany), sectioned (5 µm), and stained with H&E. The sections were scanned using an Aperio CS ScanScope, and the digitized images were used to measure seminiferous cord area. The testis slides were then scored under a microscope for the presence of MNGs; the number of MNGs per testis was normalized by seminiferous cord area and then pooled by litter.

We examined testosterone production in the fetal testis by incubating single fetal testes in M-199 media at 37°C for 3 hr. The media supernatant was collected in aliquots and analyzed for testosterone by immunoassay (Ligand Assay and Analysis Core, Center for Research in Reproduction, University of Virginia, Charlottesville, VA).

*Deiodinase inhibition*. We investigated the effects of TBMEHP on deiodinase activity using a competitive substrate assay developed by Butt et al. (2011). Briefly, rat liver microsomes (Invitrogen, Carlsbad, CA) were diluted to 1 mg protein/mL in 0.1 M potassium phosphate buffer (pH 7.4) containing dithiothreitol and NADPH. A specific mass (100 nM) of T4 was added to the samples and incubated at 37°C for 60 min in a shaking water bath with increasing concentrations (0–200 mg/mL) of TBMEHP. We selected substrate and protein concentrations such that the concentration of T4 was << *K*_m_ (Visser et al. 1979). The conversion of T4 to its deiodinated metabolites [T3, 3,3´,5´-reverse triiodothyronine (rT3), 3,3´-diiodothyronine (3,3´-T2), and 3´-monoiodothyronine] was measured using LC/MS/MS as described by Butt et al. (2011). All samples were run in triplicate. Half maximal inhibitory concentration (IC_50_) values were obtained using the one-site competition model in SigmaPlot (version 9.01; Systat Software Inc., Chicago, IL).

*Cell culture*. We purchased NIH 3T3 L1 preadipocyte cells from ATCC (Manassas, VA). FAO cells stably transfected with mouse PPARα (FAO-PPARα cells; Shipley et al. 2004) were kindly provided by D. Waxman (Boston University, Boston, MA). Stocks of NIH 3T3 L1 cells were maintained in DMEM (Dulbecco’s modified Eagle medium) with 10% calf serum (Sigma-Aldrich). Stocks of FAO-PPARα cells were maintained in DMEM with 5% fetal bovine serum (FBS) and 2 μg/mL puromycin (both from Sigma-Aldrich). Maintenance media were also supplemented with 5 μg/mL plasmocin (Invitrogen, San Diego, CA) and 20 mM l-glutamine (Mediatech, Manassas, VA). Cultures were maintained at 37°C in a humidified 5% CO_2_ atmosphere. For experiments, NIH 3T3 L1 cells and FAO-PPARα cells were plated at 40,000 cells/well (24-well plates) or 200,000 cells/well (6-well plates) in DMEM with 5% FBS, plasmocin and l-glutamine. Sodium pyruvate (1 mM; MP Biomedicals LLC, Solon, OH) was also included in NIH 3T3 L1 experimental medium. Cultures were allowed to become confluent over 3–4 days.

Prior to dosing, the medium was replaced and supplemented with insulin (0.5 μg/mL) and dexamethasone (10^–9^ M). Cultures received no treatment (naive) or were treated with vehicle (DMSO, 0.1%), the PPARγ ligand rosiglitazone (100 nM), the PPARγ ligand GW7647 (100 nM), MEHP (10–100 μM), TBPH (10–100 μM) or TBMEHP (10–100 μM). After treatment, cells were cultured for 24 hr (for mRNA expression) or 7 days (for lipid accumulation and perilipin expression). For perilipin expression experiments, medium was changed and the cultures were re-dosed on day 4. All other experiments received a single treatment.

For transduction, lentiviral particles were prepared with the MISSION™ Non-target shRNA (short hairpin RNA) control vector (SHC002) or a MISSION™ PPARγ shRNA vector (TRCN0000001658 or TRCN0000001660; Sigma-Aldrich), as previously described (Yanik et al. 2011). Medium on established NIH 3T3 L1 cultures was replaced with medium containing 8 μg/mL polybrene. Cultures were not transduced or they were transduced with either the nontarget lentivirus [multiplicity of infection (MOI) of 30:1] or with PPARγ-shRNA lentivirus (MOI of 15:1 for each virus) and incubated for 3 days. Before dosing, the medium was replaced with medium containing 10 nM dexamethasone and 0.5 μg/mL insulin. Cultures received no treatment (naive) or were treated with vehicle (DMSO, 0.1%) or TBMEHP (80 μM). On day 3, medium was replaced and the cultures were re-treated. Cells were harvested on days 4 and 7 of treatment.

To determine lipid accumulation, cells were washed once in cold phosphate-buffered saline (PBS) and stained with an aqueous solution of Nile Red (1 μg/mL in PBS) for 10 min (Greenspan et al. 1985). Fluorescence [excitation 485 (20-nm bandwidth), emission 530 nm (25-nm bandwidth)] was measured using a Synergy2 multifunction plate reader (BioTek, Winooski, VT). Fluorescence in the experimental wells was normalized by subtracting the fluorescence measured in wells containing naive cells.

To determine perilipin expression, cells were washed once in cold PBS, collected, lysed in Cell Lysis Buffer (Cell Signaling Technology, Beverly, MA), and sonicated. The lysates were cleared by centrifugation, and the supernatants were used for protein expression analyses. Protein concentrations were determined by the Bradford method (Bradford 1976). Total proteins (15–45 μg) were resolved on 10% gels, transferred to a 0.2-µm nitrocellulose membrane, and incubated with polyclonal rabbit anti-perilipin (catalog no. 3470; Cell Signaling Technology). Immunoreactive bands were detected using horseradish peroxidase–conjugated secondary antibodies (Biorad, Hercules, CA) followed by electrochemiluminescence. To control for equal protein loading, blots were re-probed with a β-actin–specific antibody (catalog no. A5441; Sigma-Aldrich) and analyzed as described above.

To determine changes in mRNA expression, cells were washed once in cold PBS and frozen at –80°C. Total RNA was extracted and genomic DNA was removed using the RNeasy Plus Mini Kit (Qiagen, Valencia, CA). cDNA was prepared using the GoScript™ Reverse Transcription System (Promega, Madison, WI), with a 1:1 mixture of random and oligo (dT)_15_ primers. Real-time quantitative polymerase chain reaction (qPCR) was performed using the GoTaq® qPCR Master Mix System (Promega). The following validated primers were purchased from Qiagen Inc.: mouse *PPAR*γ *1/2* [Genbank accession no. NM_011146 (http://www.ncbi.nlm.nih.gov/genbank/)], mouse *FABP4* (fatty acid binding protein 4; NM_024406), mouse *18S RNA* (NR_003278), rat *AOX* (acyl co-A oxidase; NM_017340), and rat *18S/28S RNA* (M11188). qPCR reactions (in duplicate) were performed using an Applied Biosystems 7300 Real-Time PCR System (Applied Biosystems, Carlsbad, CA) as follows: hot-start activation at 95°C for 2 min, 40 cycles of denaturation (95°C for 15 sec), and annealing/extension (55°C for 60 sec). Relative gene expression was determined using the comparative C_T_ method, using the threshold value for 18S RNA or 18S/28S RNA for normalization. The C_T_ value for untreated samples was used as the reference point.

*Statistics*. All statistical analyses were performed using using GraphPad Prism 5.0 (GraphPad Software, La Jolla, CA); differences between treatment groups were considered statistically significant at *p* < 0.05. Data from clinical chemistry, quantitative values from histopathology and immunohistochemistry, and *in vitro* lipid accumulation and gene expression data were analyzed by one-way analysis of variance (ANOVA) followed by Dunnett’s post hoc test. Data from transduction experiments were analyzed by two-way ANOVA followed by the Bonferroni post hoc test. Normality or log-normality of dust concentration data were assessed using histograms and Shapiro–Wilks tests. Because dust data were neither log-normal nor normal, concentrations in different microenvironments are reported using medians and were compared using nonparametric Wilcoxon rank sum tests.

## Results

*Exposure*. Median concentrations of TBPH in dust were 150 ng/g (range, < 4–12,400 ng/g) in main living areas of homes (*n* = 31; 74% > LOD), 410 ng/g (range, 95–15,500 ng/g) in offices (*n* = 31; 100% > LOD), and 400 ng/g (range, < 36–4,830 ng/g) in cars (*n* = 20; 90% > LOD). TBPH concentrations of offices and cars were significantly different from those of homes (*p* < 0.05).

*TBPH metabolism*. *In vitro* metabolism of TBPH in the presence of hepatic porcine esterase resulted in the formation of TBMEHP at a rate of 89 pmol/hr/mg esterase.

**Figure 2:**
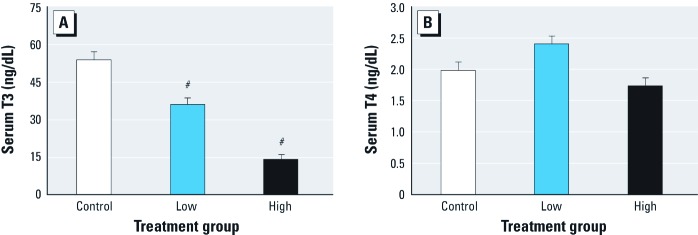
Changes in serum T3 (*A*) and T4 (*B*) levels (mean ± SE) in dams treated with low (200 mg/kg) or high (500 mg/kg) doses of TBMEHP on GD18 and GD19. T3 levels exhibited a statistically significant dose-dependent decrease after TBMEHP exposure (*A*); however, T4 levels were not significantly altered (*B*). *n *= 10 vehicle controls, 9 in the low-dose group, and 10 in the high-dose group. ^#^*p *< 0.001 compared with vehicle controls.

In vivo *rat experiments*. In pregnant Fischer rats gavaged daily for 2 days (GD18 and GD19) with TBMEHP, we found no significant differences in liver, kidney, adrenal gland, and ovary weights compared with vehicle controls. Results of maternal serum clinical chemistries are presented in Table 1. In dams that received the high TBMEHP dose (500 mg/kg), the liver enzyme alkaline phosphatase was significantly decreased but alanine transaminase was significantly increased. Also in the high-dose group, blood urea nitrogen levels were significantly higher and calcium levels were significantly lower. Cholesterol levels decreased significantly in a dose-dependent manner. Serum T3 was significantly reduced in a dose-dependent-manner, but we observed no effect on serum T4 (Figure 2).

**Table 1:** Clinical chemistry parameters from TBMEHP-exposed dams (mean ± SE).

Analyte	Control	Low dose (200 mg/kg)	High dose (500 mg/kg)
Blood urea nitrogen (mg/dL)	13.90 ± 0.46	12.11 ± 0.59	18.50 ± 1.28**
Calcium (mg/dL)	10.96 ± 0.13	10.56 ± 0.15	10.34 ± 0.11**
Alkaline phosphatase (mg/dL)	254.0 ± 8.7	273.6 ± 12.1	189.9 ± 10.0#
Alanine transaminase (IU/L)	62.00 ± 1.96	62.11 ± 3.43	81.70 ± 2.29#
Cholesterol (mg/dL)	55.70 ± 1.14	47.89 ± 1.73**	30.00 ± 1.92#
Glucose (mg/dL)	96.70 ± 5.64	99.25 ± 2.83	93.40 ± 7.04
Sodium (mEq/L)	138.2 ± 0.7	137.3 ± 0.6	138.3 ± 0.4
Potassium (mmol/L)	5.830 ± 0.097	5.656 ± 0.162	5.890 ± 0.211
Chloride (mmol/L)	94.40 ± 0.60	92.89 ± 0.68	94.90 ± 1.04
Carbon dioxide (mEq/L)	27.90 ± 0.43	28.78 ± 0.55	26.90 ± 1.48
Creatinine (mg/dL)	0.3040 ± 0.0156	0.3289 ± 0.0203	0.3530 ± 0.0189
Total protein (g/dL)	5.080 ± 0.096	4.944 ± 0.075	5.190 ± 0.080
Albumin (g/dL)	1.430 ± 0.034	1.389 ± 0.035	1.420 ± 0.013
Phosphorous (mg/dL)	11.16 ± 0.22	10.84 ± 0.31	10.88 ± 0.36
Uric acid (mg/dL)	2.04 ± 0.19	1.73 ± 0.17	1.98 ± 0.19
Aspartate transaminase (IU/L)	87.2 ± 6.9	89.0 ± 7.9	108.6 ± 9.9
Triglycerides (mg/dL)	188.9 ± 18.8	301.0 ± 40.8	267.2 ± 40.7
**p < 0.01, and #p < 0.001 compared with control by ANOVA followed by Dunnett’s post hoc test.						

**Figure 3:**
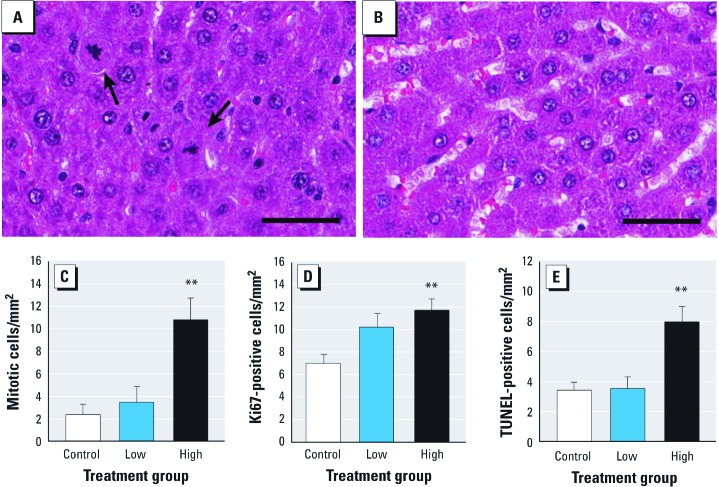
Mitotic activity, proliferation, and apoptosis of hepatocytes from dams exposed to TBMEHP or vehicle on GD18 and GD19. (*A,B*) Histopathology of H&E-stained liver sections (*A*) from a high-dose dam, and (*B*) from a vehicle control dam. Liver sections are stained with H&E; bar = 50 µm. Arrows indicate hepatocytes undergoing mitosis. An increase in mitotic activity was observed at the high dose both by quantifying mitotic cells by histopathology (*C*) and by assessing positive Ki67 cells by immunohistochemistry (*D*). (*E*) TUNEL staining resulted in a significant increase in apoptotic hepatocytes at the high dose. Results are presented as mean ± SE. *n*= 10 vehicle controls; 9 in the low-dose group, and 10 in the high-dose group. **p < 0.01 compared with vehicle controls.

Given the effects on dam livers, kidneys, and thyroids indicated by the serum clinical chemistries, we evaluated these organs histopathologically. In TBMEHP-exposed dams, we observed no abnormalities in the kidneys or thyroids, whereas the livers were abnormal. Qualitatively, liver sections from TBMEHP-exposed dams showed an increase in hepatocytes with mitotic spindles (Figure 3A,B), indicating proliferation, and an increase in hepatocytes with dense hypereosinophilic cytoplasm and condensed, fragmented nuclei characteristic of apoptosis. These qualitative impressions were confirmed by quantifying the number of cells in mitosis across groups, with a significant increase in mitotic activity observed in high-dose dams (Figure 3C). Using the proliferation marker Ki67, we quantified the number of proliferating hepatocytes and found a significant increase in proliferation in the high-dose group (Figure 3D). The qualitative impression of TBMEHP-induced hepatocyte apoptosis was confirmed by TUNEL staining of liver cross-sections, with high-dose dams having a significant increase in TUNEL-positive events (Figure 3E).

**Figure 4:**
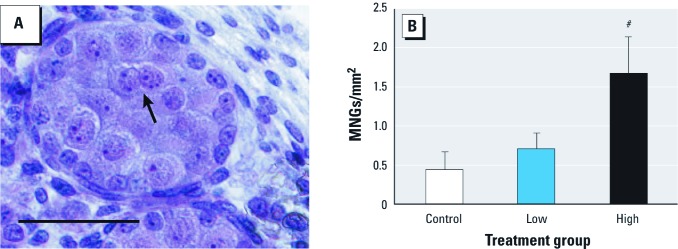
Induction of MNGs in fetal testes from rats exposed prenatally (GD18 and GD19) to TBMEHP or vehicle. (*A*) MNG (arrow) in an H&E-stained cross-section of a fetal seminiferous cord after high-dose TBMEHP exposure (bar = 50 µm). (*B*) MNGs were significantly increased after TBMEHP exposure (normalized to seminiferous cord area). Results are presented as mean ± SE. *n *= 10 vehicle controls, 9 in the low-dose group, and 10 in the high-dose group. *^#^p *< 0.001 compared with vehicle controls.

Because of the known effects on fetal testes of MEHP, the nonbrominated analog of TBMEHP (Figure 1), we evaluated testes from fetuses of exposed dams for induction of MNGs (a manifestation of altered seminiferous cords) and testosterone production. Cross-sections of fetal testes were scored for the number of MNGs (Figure 4A); in high-dose fetal testes we found a significantly increased number of MNGs per cord area (Figure 4B). After *ex vivo* incubation of fetal testes in media, we observed no significant changes in testosterone production in treated animals compared with controls (control, 1.47 ± 0.06 μg/dL; low dose, 1.36 ± 0.11 μg/dL; high dose, 1.28 ± 0.05 μg/dL).

**Figure 5:**
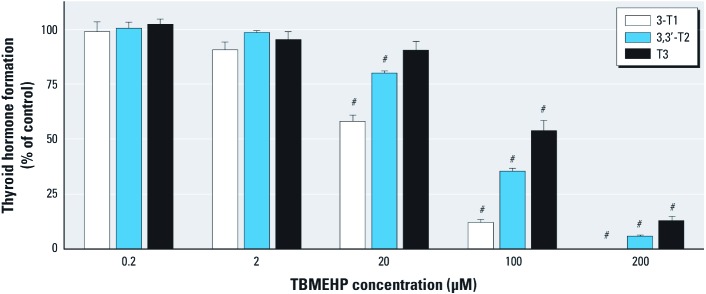
Inhibition of deiodinase activity by TBMEHP in rat liver microsomes. A dose-dependent decrease in the levels of deiodinated T4 metabolites—3´-monoiodothyronine (3‑T1), 3,3´‑diiodothyronine (3,3´‑T2), and T3—was observed using LC/MS/MS. Data (mean ± SE) are representative of three independent experiments. *^#^p *< 0.001 compared with vehicle controls.

*Deiodinase activity*. Co-incubation of TBMEHP and T4 with rat hepatic microsomes resulted in a dose-dependent decrease in deiodinase activity (Figure 5). The conversion of T4 to T3 by outer-ring deiodinase (ORD) activity was significantly inhibited in the presence of TBMEHP, and the IC_50_ value of ORD activity was approximately 132 μM. T3 can be further metabolized by deiodinases to 3,3´-T2 via inner-ring deiodination (IRD) activity. TBMEHP also inhibited the formation of 3,3´-T2 from T4 metabolism with an IC_50_ of approximately 78 μM. Further metabolism of 3,3´-T2 to 3-T1 by ORD activity was also inhibited by TBMEHP, with an IC_50_ of 35 μM. We did not observe the formation of rT3 by IRD activity on T4 (data not shown).

**Figure 6:**
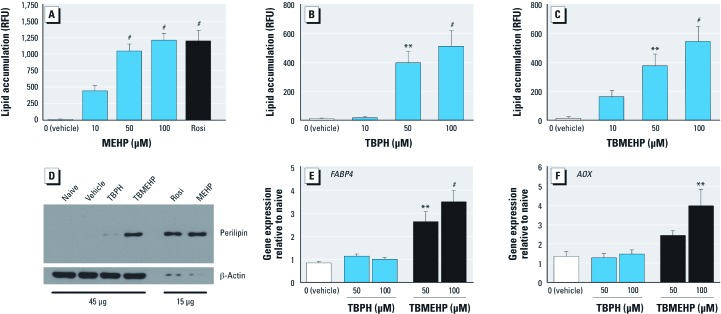
*In vitro* activation of PPARα and PPARγ by TBPH and TBMEHP. Cell lysates from NIH 3T3 L1 cells treated for 7 days with MEHP (50–100 μM), or rosiglitazone (Rosi; 100 nM), TBPH, TBMEHP or 0.1% DMSO (vehicle) in the presence of insulin (0.5 μg/mL) and dexamethasone (10^–9^ M) were stained with Nile Red and their fluorescence measured to quantify lipid accumulation (*A–C*) and immunoblotted for perilipin expression (*D*). RFU, relative fluorescence units. Gene expression analysis of *FABPB4* in NIH 3T3 L1 cells (*E*) and *AOX* in FAO-PPARα cells (*F*) treated with TBPH or TBMEHP for 24 hr was performed using qPCR. Data (mean ± SE) are representative of at least three independent experiments. **p < 0.01, and ^#^*p *< 0.001 compared with vehicle controls.

*Cell culture*. Given the ability of MEHP to activate both PPARγ and PPARα (Bility et al. 2004; Feige et al. 2007; Hurst and Waxman 2003), we hypothesized that TBMEHP is a PPAR ligand. To test the ability of TBPH and TBMEHP to activate PPARγ-dependent adipocyte differentiation, we treated murine NIH 3T3 L1 cells with TBPH or TBMEHP for 7 days and compared them to cells treated with MEHP, rosiglitazone, or vehicle. The positive controls (rosiglitazone and MEHP) were highly efficient at stimulating adipocyte differentiation, as indicated by significant lipid accumulation after exposure to 50 μM or 100 μM, respectively (Figure 6A).

Surprisingly, both TBPH and TBMEHP stimulated lipid accumulation (Figure 6B,C); however, although their potency was similar to that of MEHP, the brominated phthalates had a lower efficacy for stimulating lipid accumulation. To confirm that TBPH and TBMEHP stimulated terminal adipocyte differentiation, we assessed NIH 3T3 L1 cells for expression of the adipocyte-specific protein perilipin (Ducharme and Bickel 2008). Treatment with TBMEHP resulted in an increased expression of perilipin, whereas TBPH only minimally increased expression of perilipin (Figure 6D). Consistent with the lower efficacy of TBMEHP to stimulate lipid accumulation, the extent of perilipin up-regulation was less in TBMEHP-treated cells than in MEHP- or rosiglitazone-treated cells. These results indicate that TBMEHP stimulates adipogenesis, a PPARγ-dependent process, in a classic preadipocyte model, although this model has a limited efficacy.

To investigate the potential for the brominated phthalates to stimulate PPAR-driven transcription, we measured expression of the PPAR-mediated genes *FABP4* (PPARγ target) (Tontonoz et al. 1994) and *AOX* (PPARα target) (Reddy and Hashimoto 2001) in treated NIH 3T3 L1 and FAO-PPARα cells, respectively (Figure 6E,F). Cells were treated for 24 hr to assess early, receptor-dependent gene expression responses. TBMEHP, but not TBPH, significantly up-regulated the expression of both *FABP4* and *AOX* mRNA. Similar to the results seen in the adipocyte differentiation assay, TBMEHP had a lower efficacy for stimulating PPARγ and PPARα transactivation than did MEHP. MEHP (100 μM) exposure resulted in an 11.5 ± 2.7 fold increase in *FABP* expression and a 5.4 ± 0.6 fold increase in *AOX* expression, similar to the positive controls rosiglitazone (*FABP4*: 6.0 ± 1.4 fold increase) and GW7647 (*AOX*: 4.9 ± 0.5 fold increase).

**Figure 7:**
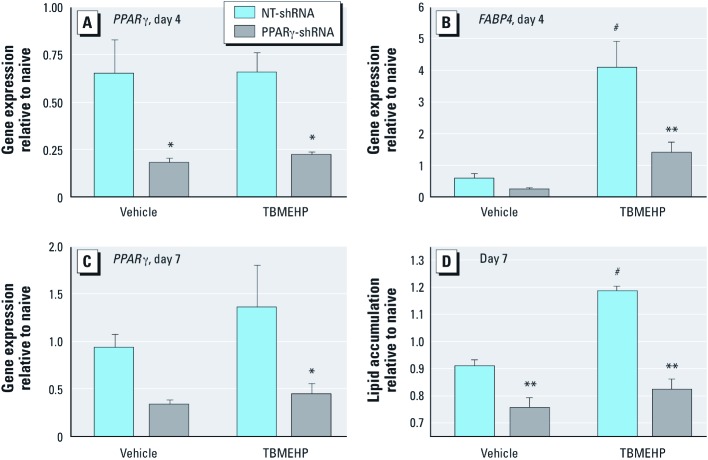
Gene expression of *PPAR*γ (*A*), *FABP4* (*B*), and *PPAR*γ (*C*), and lipid accumulation (*D*) in NIH 3T3 L1 cells transduced with non-target (NT)-shRNA or PPARγ-shRNA and treated with TBMEHP (80 μM) or vehicle (0.1% DMSO) for 4 (*A,B*) or 7 (*C,D*) days. Data are presented as mean ± SE from at least three independent experiments. *p < 0.05, and **p < 0.01 compared with NT‑shRNA. ^#^*p *< 0.01 compared with vehicle controls.

We tested the contribution of PPARγ to TBMEHP-induced differentiation in NIH 3T3 L1 cells by knocking down *PPAR*γ expression using lentivirus–delivered PPARγ-shRNA. Expression of *PPAR*γ was significantly reduced in transduced cells, compared with those transduced with a nontarget construct on day 4 of treatment (Figure 7A). This was evident in both vehicle- and TBMEHP-treated cells. Furthermore, TBMEHP-induced expression of the PPARγ target gene, *FABP4*, was significantly impaired in PPARγ-shRNA transduced cells (Figure 7B). Reduction of *PPAR*γ expression remained evident at 7 days (Figure 7C), and TBMEHP-induced lipid accumulation was significantly suppressed in PPARγ-shRNA transduced cells (Figure 7D). These results indicate that TBMEHP is a ligand for PPARs and is capable of stimulating PPARγ-dependent differentiation.

## Discussion

The novel flame retardant TBPH has been in use since 2003 and is already present in the environment and in the tissues of some mammals (Lam et al. 2009). Although the risk for human exposure clearly exists, the possible health effects of TBPH and its metabolites are poorly understood. Given the lack of existing information, this study was designed to provide insight into routes and extent of exposure and TBPH metabolites of potential concern. Because TBPH is structurally similar to DEHP and because the monoester phthalates are known to be the major toxic phthalate metabolites, we focused particular attention on the TBPH metabolite TBMEHP. We assessed the toxicity of TBMEHP in pregnant rats and conducted *in vitro* mechanistic studies on this metabolite.

Our data demonstrate the presence of TBPH at levels up to parts per million in dust from homes, offices, and cars, three of the indoor microenvironments most likely to lead to human exposure. Exposure may take place via hand-to-mouth activity (of particular concern for children), although dermal absorption and inhalation of suspended particles may also be possible. Associations between levels in dust, handwipes, and human body burdens (serum or breast milk) have been previously demonstrated for pentaBDEs, the fire retardant compounds that Firemaster 550 replaced (Watkins et al. 2011; Wu et al. 2007). TBPH has not yet been measured in humans. Median TBPH dust concentrations were approximately three times as high in office dust as in homes. This difference may be due to several factors. For example:

Many offices in this study contained foam furniture purchased since 2004, when pentaBDE was phased out of production and replaced by Firemaster 550 and other flame retardants.
Foam furniture used in offices in Boston is generally required to meet California fire retardant standards, but this does not apply to use in homes.
Use of wires and cables treated with DP-45, another TBPH-containing flame retardant, has increased in offices (Stapleton et al. 2008).

TBPH has not been previously reported in dust from offices or automobiles. In 2006, dust from the main living areas of 16 other Boston homes were sampled and analyzed for TBPH using the same collection methods and laboratory (Stapleton et al. 2008). Median TBPH concentrations were 330 ng/g in the 2006 samples and 150 ng/g in the 2009 samples (150 ng/g), but caution is needed in comparing these results because of some differences in extraction methods.

Understanding the metabolism of TBPH is critical to predicting its toxicity. Without further information on the pharmacokinetics of TBPH and its metabolites, it is difficult to compare potential human exposure to this compound via dust with the doses of TBMEHP used in our rat study. Nonenzymatic hydrolysis of phthalate esters is primarily base-catalyzed (Steinberg and Lena 1995), indicating that the breakdown of phthalate esters takes place in the intestines rather than in the highly acidic stomach environment. The electron-withdrawing effects of the bromine groups added to the aromatic ring could speed up alkaline hydrolysis; alternatively, steric hindrance by the large bromine groups could cause hydrolysis of TBPH to occur more slowly than for DEHP.

Esterase activity plays a major role in the metabolism of the TBPH analog DEHP (Agency for Toxic Substances and Disease Registry 2002; Koch et al. 2006). In rats, DEHP is hydrolyzed to MEHP in the intestines via the action of gut esterases (Albro 1986; Albro et al. 1982; Barber et al. 1994). However, at DEHP doses of 100 mg/kg in rats, DEHP can be measured in the feces, representing unabsorbed compound that is unavailable for hydrolysis to MEHP (Keys et al. 1999). There is limited information regarding the *in vivo* metabolism of TBPH, however, its structural similarity to DEHP suggests that TBPH could be metabolized in an analogous manner. The *in vitro* formation of TBMEHP observed in this study provides evidence that mammalian esterases are capable of mediating the hydrolysis of TBPH, and that exposure to TBMEHP may occur as a result of *in vivo* exposure to TBPH. However, the formation rate of TBMEHP (89 pmol/hr/mg esterase) was much slower than the rapid formation of MEHP from DEHP, the nonbrominated analog of TBPH, observed in a previous study with porcine hepatic esterase (7.6 nmol/hr/mg esterase) (Niino et al. 2003). Therefore, metabolism of TBPH to TBMEHP likely can occur *in vivo*, but further work is necessary to investigate the metabolism of TBPH in other important tissues, such as intestinal cells and serum, and to measure the rate at which it occurs *in vivo*.

*In utero* MEHP exposure causes antiandrogenic effects in the rat fetal testis, demonstrating that this critical window of development is sensitive to toxic insult. To that end, we examined whether *in utero* exposure to TBMEHP (on GD18 and GD19) induced antiandrogenic effects. TBMEHP, unlike MEHP, did not reduce testosterone production by the fetal testis and thus does not appear to share the antiandrogenic action of its nonbrominated analog. Exposure of the fetal testes to TBMEHP induced MNGs, an effect previously observed following exposure to MEHP and other active phthalates (Foster 2006; Gaido et al. 2007; Gray et al. 2006; Heger et al. 2012; Lehraiki et al. 2009). MNG formation is a convenient measure of phthalate-induced effects on the seminiferous cords (Boekelheide et al. 2009; Howdeshell et al. 2008).

In dams exposed to TBMEHP, serum clinical chemistries showed changes in liver enzyme levels, indicating damage to the liver. Histopathological examination and staining for Ki67 and TUNEL in liver from these dams confirmed the presence of hepatocyte apoptosis and proliferation. Although the underlying mechanisms of TBMEHP-induced hepatocyte damage and proliferation remain to be determined, one contributing factor is likely to be its PPARα agonist activity, which we identified in cell culture models. Peroxisome proliferators, such as DEHP, are known to exert their hepatotoxic effects through activation of PPARα, as demonstrated by a lack of DEHP-induced peroxisome proliferation, hepatic enlargement, and increases in peroxisomal enzyme expression in PPARα-null mice (Ward et al. 1998).

The significant, dose-dependent decrease in dam serum T3 may be attributed to TBMEHP acting as a deiodinase inhibitor, preventing conversion of T4 to T3 in peripheral tissues. Thyroid-stimulating hormone causes the release of T4 after its formation from thyroglobulin in the thyroid. T4 is then deiodinated by enzymes to produce T3, both within the thyroid gland itself and more extensively in the peripheral tissues. Inhibition of the deiodinase enzymes by xenobiotics is a well-characterized pathway of toxicity (Butt et al. 2011; Ferreira et al. 2002; Schmutzler et al. 2004). Deiodinase inhibitors lead to a rapid decrease in serum T3 levels, followed by a more gradual increase in T4. The results of the deiodinase assay indicate that TBMEHP acts as an ORD inhibitor, preventing conversion of T4 to T3. Butt et al. (2011) observed a similar pattern of ORD inhibition by several classes of halogenated phenolic compounds. The TBMEHP IC_50_ values we observed are similar to those observed for 2,4,6-trichlorophenol and for a fluorinated analog of bisphenol A (Butt et al. 2011). However, disruption of thyroid hormones can also arise from the inhibition of sulfotransferases (Schuur et al. 1998a, 1998b), suggesting that TBMEHP may affect T3 and T4 levels via multiple mechanisms. Even though we found a highly significant effect of TBMEHP on serum T3 levels in the present study, histopathological examination of thyroid sections revealed no abnormalities. This is likely because the dosing period of 2 days is too short to produce histological changes in the thyroid. In a study involving exposure of rats to PBDEs and polychlorinated biphenyls, 14 consecutive days of treatment was sufficient to produce observable changes in thyroid histopathology (Hallgren and Darnerud 2002).

Impaired thyroid activity is associated with levels of urinary MEHP, the unbrominated analog of TBMEHP. An epidemiological study of the general U.S. population found a negative association between MEHP concentrations and serum T3 levels (Meeker et al. 2007). These results are consistent with our finding of a dose-dependent decrease in serum T3 levels induced by TBMEHP. Extrapolation of these findings in combination with the epidemiological MEHP evidence suggests that TBMEHP may disrupt thyroid function in humans in a manner similar to that of its unbrominated analog, MEHP.

Our *in vitro* mechanistic experiments revealed that TBMEHP activates both PPARα- and PPARγ-mediated gene transcription and is capable of stimulating PPARγ-mediated adipocyte differentiation. These results are in accordance with previous studies that demonstrated that the nonbrominated phthalate monoester MEHP is a ligand for both PPARα and PPARγ (Bility et al. 2004; Feige et al. 2007; Hurst and Waxman 2003). These interactions open the possibility for TBMEHP to exert toxic effects through superphysiological activation of these nuclear receptors. As indicated above, dam hepatotoxicity likely resulted from TBMEHP-driven activation of PPARα (Ward et al. 1998), and this exposure could also result in peroxisome proliferation and aberrant fatty acid metabolism (Aoyama et al. 1998; Reddy and Hashimoto 2001). The ability of TBMEHP to activate PPARγ and stimulate adipocyte differentiation places it in an emerging class of environmental contaminants, the environmental obesogens; this class also includes phthalates, organotins, and brominated bisphenol A (Feige et al. 2007; Grün et al. 2006; Riu et al. 2011). Indeed, phthalates and organotins have been shown to increase fat accumulation *in vivo* (Feige et al. 2010; Grün et al. 2006). Overall, the activation of PPARγ by TBMEHP, as well as the subsequent downstream detrimental physiological responses, underscores the need to identify and investigate the mechanisms of action of compounds in this toxicant class.

## Conclusions

In the present study, dust collected from homes, offices, and cars was shown to contain relatively large (parts per million) amounts of TBPH, implying human exposure via contact with dust and hand-to-mouth activity (particularly important for children) and potentially via other pathways. Metabolic experiments identified the TBPH monoester metabolite, TBMEHP, as a product of mammalian esterase activity. TBMEHP was toxicologically assessed by exposing pregnant rat dams to 500 mg/kg or 200 mg/kg TBMEHP or to vehicle. After 2 days of exposure (GD18 and GD19), serum liver enzyme levels of the dams were altered, and histopathological analysis of dam livers showed hepatocyte apoptosis and proliferation. We observed that serum T3 levels were significantly decreased in a dose-dependent manner and that TBMEHP inhibited deiodinase activity. Testes isolated from the fetuses of exposed dams showed induction of MNGs in the high-dose group without significant effects on testosterone production. In *in vitro* mechanistic studies using murine FAO and NIH 3T3 L1 cells, we identified TBMEHP as a PPARα and PPARγ agonist. The results of our experiments using TBMEHP exposure in rodent *in vitro* and *in vivo* models, along with the increasing environmental levels of TBPH, identify alterations in fetal testis development, thyroid dysfunction, liver dysfunction, and obesity as potential concerns with these chemicals.

## References

[r1] Agency for Toxic Substances and Disease Registry (2002). Toxicological Profile for Di(2-ethylhexyl)phthalate (DEHP). Atlanta, GA:Agency for Toxic Substances and Disease Registry.. http://www.atsdr.cdc.gov/toxprofiles/tp9.pdf.

[r2] Albro PW (1986). Absorption, metabolism, and excretion of di(2-ethylhexyl) phthalate by rats and mice.. Environ Health Perspect.

[r3] Albro PW, Hass JR, Peck CC, Jordan ST, Corbett JT, Schroeder J (1982). Applications of isotope differentiation for metabolic studies with di-(2-ethylhexyl) phthalate.. J Environ Sci Health B.

[r4] Allen JG, McClean MD, Stapleton HM, Webster TF (2008). Critical factors in assessing exposure to PBDEs via house dust.. Environ Int.

[r5] Aoyama T, Peters JM, Iritani N, Nakajima T, Furihata K, Hashimoto T (1998). Altered constitutive expression of fatty acid-metabolizing enzymes in mice lacking the peroxisome proliferator-activated receptor α (PPARα).. J Biol Chem.

[r6] Barber ED, Fox JA, Giordano CJ (1994). Hydrolysis, absorption and metabolism of di(2-ethylhexyl) terephthalate in the rat.. Xenobiotica.

[r7] Bility MT, Thompson JT, McKee RH, David RM, Butala JH, Vanden Heuvel JP (2004). Activation of mouse and human peroxisome proliferator-activated receptors (PPARs) by phthalate monoesters.. Toxicol Sci.

[r8] Birnbaum LS, Staskal DF (2004). Brominated flame retardants: cause for concern?. Environ Health Perspect.

[r9] Bloom M, Spliethoff H, Vena J, Shaver S, Addink R, Eadon G. (2008). Environmental exposure to PBDEs and thyroid function among New York anglers.. Environ Toxicol Pharmacol.

[r10] Boekelheide K, Kleymenova E, Liu K, Swanson C, Gaido KW (2009). Dose-dependent effects on cell proliferation, seminiferous tubules, and male germ cells in the fetal rat testis following exposure to di(*n*-butyl) phthalate.. Microsc Res Tech.

[r11] Bradford MM (1976). A rapid and sensitive method for the quantitation of microgram quantities of protein utilizing the principle of protein-dye binding.. Anal Biochem.

[r12] Butt CM, Wang D, Stapleton HM (2011). Halogenated phenolic contaminants inhibit the *in vitro* activity of the thyroid-regulating deiodinases in human liver.. Toxicol Sci.

[r13] Costa LG, Giordano G, Tagliaferri S, Caglieri A, Mutti A (2008). Polybrominated diphenyl ether (PBDE) flame retardants: environmental contamination, human body burden and potential adverse health effects.. Acta Biomed.

[r14] Covaci A, Harrad S, Abdallah MA-E, Ali N, Law RJ, Herzke D (2011). Novel brominated flame retardants: a review of their analysis, environmental fate and behaviour.. Environ Int.

[r15] Darnerud PO, Eriksen GS, Jóhannesson T, Larsen PB, Viluksela M (2001). Polybrominated diphenyl ethers: occurrence, dietary exposure, and toxicology.. Environ Health Perspect.

[r16] de Wit CA (2002). An overview of brominated flame retardants in the environment.. Chemosphere.

[r17] Ducharme NA, Bickel PE (2008). Lipid droplets in lipogenesis and lipolysis.. Endocrinology.

[r18] Feige JN, Gelman L, Rossi D, Zoete V, Métivier R, Tudor C (2007). The endocrine disruptor monoethyl-hexyl-phthalate is a selective peroxisome proliferator-activated receptor γ modulator that promotes adipogenesis.. J Biol Chem.

[r19] Feige JN, Gerber A, Casals-Casas C, Yang Q, Winkler C, Bedu E (2010). The pollutant diethylhexyl phthalate regulates hepatic energy metabolism via species-specific PPARα-dependent mechanisms.. Environ Health Perspect.

[r20] Ferreira ACF, Lisboa PC, Oliveira KJ, Lima LP, Barros IA, Carvalho DP (2002). Inhibition of thyroid type 1 deiodinase activity by flavonoids.. Food Chem Toxicol.

[r21] Foster PMD (2006). Disruption of reproductive development in male rat offspring following *in utero* exposure to phthalate esters.. Int J Androl.

[r22] Gaido KW, Hensley JB, Liu D, Wallace DG, Borghoff S, Johnson KJ (2007). Fetal mouse phthalate exposure shows that gonocyte multinucleation is not associated with decreased testicular testosterone.. Toxicol Sci.

[r23] Gray LE, Wilson VS, Stoker T, Lambright C, Furr J, Noriega N (2006). Adverse effects of environmental antiandrogens and androgens on reproductive development in mammals.. Int J Androl.

[r24] Greenspan P, Mayer EP, Fowler SD (1985). Nile red: a selective fluorescent stain for intracellular lipid droplets.. J Cell Biol.

[r25] Grün F, Watanabe H, Zamanian Z, Maeda L, Arima K, Cubacha R (2006). Endocrine-disrupting organotin compounds are potent inducers of adipogenesis in vertebrates.. Mol Endocrinol.

[r26] Hagmar L, Björk J, Sjödin A, Bergman A, Erfurth EM (2001). Plasma levels of persistent organohalogens and hormone levels in adult male humans.. Arch Environ Health.

[r27] Hallgren S, Darnerud PO (2002). Polybrominated diphenyl ethers (PBDEs), polychlorinated biphenyls (PCBs) and chlorinated paraffins (CPs) in rats-testing interactions and mechanisms for thyroid hormone effects.. Toxicology.

[r28] Heger NE, Hall SJ, Sandrof MA, McDonnell EV, Hensley JB, McDowell EN (2012). Human fetal testis xenografts are resistant to phthalate-induced endocrine disruption.. Environ Health Perspect.

[r29] Howdeshell KL, Rider CV, Wilson VS, Gray LE (2008). Mechanisms of action of phthalate esters, individually and in combination, to induce abnormal reproductive development in male laboratory rats.. Environ Res.

[r30] Hurst CH, Waxman DJ (2003). Activation of PPARα and PPARγ by environmental phthalate monoesters.. Toxicol Sci.

[r31] Jones HB, Garside DA, Liu R, Roberts JC (1993). The influence of phthalate esters on Leydig cell structure and function in vitro and in vivo.. Exp Mol Pathol.

[r32] Julander A, Karlsson M, Hagström K, Ohlson CG, Engwall M, Bryngelsson I-L (2005). Polybrominated diphenyl ethers—plasma levels and thyroid status of workers at an electronic recycling facility.. Int Arch Occup Environ Health.

[r33] Keys DA, Wallace DG, Kepler TB, Conolly RB (1999). Quantitative evaluation of alternative mechanisms of blood and testes disposition of di(2-ethylhexyl) phthalate and mono(2-ethylhexyl) phthalate in rats.. Toxicol Sci.

[r34] Koch HM, Preuss R, Angerer J (2006). Di(2-ethylhexyl)phthalate (DEHP): human metabolism and internal exposure—an update and latest results.. Int J Androl.

[r35] Lam JCW, Lau RKF, Murphy MB, Lam PKS (2009). Temporal trends of hexabromocyclododecanes (HBCDs) and polybrominated diphenyl ethers (PBDEs) and detection of two novel flame retardants in marine mammals from Hong Kong, South China.. Environ Sci Technol.

[r36] Lehmann KP, Phillips S, Sar M, Foster PMD, Gaido KW (2004). Dose-dependent alterations in gene expression and testosterone synthesis in the fetal testes of male rats exposed to di (*n*-butyl) phthalate.. Toxicol Sci.

[r37] Lehraiki A, Racine C, Krust A, Habert R, Levacher C. (2009). Phthalates impair germ cell number in the mouse fetal testis by an androgen-and estrogen-independent mechanism.. Toxicol Sci.

[r38] Liu K, Lehmann KP, Sar M, Young SS, Gaido KW (2005). Gene expression profiling following in utero exposure to phthalate esters reveals new gene targets in the etiology of testicular dysgenesis.. Biol Reprod.

[r39] Ma Y, Venier M, Hites RA (2011). 2-Ethylhexyl tetrabromobenzoate and bis(2-ethylhexyl) tetrabromophthalate flame retardants in the Great Lakes atmosphere.. Environ Sci Technol.

[r40] Meeker JD, Calafat AM, Hauser R (2007). Di(2-ethylhexyl) phthalate metabolites may alter thyroid hormone levels in men.. Environ Health Perspect.

[r41] Meneses M, Wingfors H, Schuhmacher M, Domingo JL, Lindström G, Van Bavel B (1999). Polybrominated diphenyl ethers detected in human adipose tissue from Spain.. Chemosphere.

[r42] Niino T, Ishibashi T, Ishiwata H, Takeda K, Onodera S. (2003). Characterization of human salivary esterase in enzymatic hydrolysis of phthalate esters.. J Health Sci.

[r43] Parks LG, Ostby JS, Lambright CR, Abbott BD, Klinefelter GR, Barlow NJ (2000). The plasticizer diethylhexyl phthalate induces malformations by decreasing fetal testosterone synthesis during sexual differentiation in the male rat.. Toxicol Sci.

[r44] Reddy JK, Hashimoto T (2001). Peroxisomal β-oxidation and peroxisome proliferator-activated receptor α: an adaptive metabolic system.. Annu Rev Nutr.

[r45] Riu A, Grimaldi M, le Maire A, Bey G, Phillips K, Boulahtouf A (2011). Peroxysome proliferator-activated receptor γ is a target for halogenated analogs of bisphenol A.. Environ Health Perspect.

[r46] Roberts SC, Macaulay LJ, Stapleton HM (2012). In vitro metabolism of the brominated flame retardants 2-ethylhexyl-2,3,4,5-tetrabromobenzoate (TBB) and bis(2-ethylhexyl) 2,3,4,5-tetrabromophthalate (TBPH) in human and rat tissues.. Chem Res Toxicol.

[r47] Sahlström L, Sellström U, de Wit CA (2011). Clean-up method for determination of PBDEs, HBCDs and emerging BFRs in dust.. Organohalogen Compounds.

[r48] Schmutzler C, Hamann I, Hofmann PJ, Kovacs G, Stemmler L, Mentrup B (2004). Endocrine active compounds affect thyrotropin and thyroid hormone levels in serum as well as endpoints of thyroid hormone action in liver, heart and kidney.. Toxicology.

[r49] Schriks M, Roessig JM, Murk AJ, Furlow JD (2007). Thyroid hormone receptor isoform selectivity of thyroid hormone disrupting compounds quantified with an in vitro reporter gene assay.. Environ Toxicol Pharmacol.

[r50] Schuur AG, Legger FF, van Meeteren ME, Moonen MJH, van Leeuwen-Bol I, Bergman Å (1998a). In vitro inhibition of thyroid hormone sulfation by hydroxylated metabolites of halogenated aromatic hydrocarbons.. Chem Res Toxicol.

[r51] Schuur AG, van Leeuwen-Bol I, Jong WM, Bergman A, Coughtrie MW, Brouwer A (1998b). *In vitro* inhibition of thyroid hormone sulfation by polychlorobiphenylols: isozyme specificity and inhibition kinetics.. Toxicol Sci.

[r52] Shipley JM, Hurst CH, Tanaka SS, DeRoos FL, Butenhoff JL, Seacat AM (2004). *trans*-Activation of PPARα and induction of PPARα target genes by perfluorooctane-based chemicals.. Toxicol Sci.

[r53] Shultz VD, Phillips S, Sar M, Foster PM, Gaido KW (2001). Altered gene profiles in fetal rat testes after *in utero* exposure to di(*n*-butyl) phthalate.. Toxicol Sci.

[r54] Sjödin A, Hagmar L, Klasson-Wehler E, Kronholm-Diab K, Jakobsson E, Bergman A. (1999). Flame retardant exposure: polybrominated diphenyl ethers in blood from Swedish workers.. Environ Health Perspect.

[r55] Stapleton HM, Allen JG, Kelly SM, Konstantinov A, Klosterhaus S, Watkins D (2008). Alternate and new brominated flame retardants detected in U.S. house dust.. Environ Sci Technol.

[r56] Steinberg S, Lena F. (1995). Hydrolysis of several substituted methyl benzoates in the aqueous solution.. Water Res.

[r57] Tontonoz P, Hu E, Graves RA, Budavari AI, Spiegelman BM (1994). mPPARγ2: tissue-specific regulator of an adipocyte enhancer.. Genes Dev.

[r58] Turyk ME, Persky VW, Imm P, Knobeloch L, Chatterton R, Anderson HA (2008). Hormone disruption by PBDEs in adult male sport fish consumers.. Environ Health Perspect.

[r59] U.S. EPA 2009a Detailed Chemical Results: 1,2-Benzenedicarboxylic Acid, 3,4,5,6-Tetrabromo-, Bis(2-ethylhexyl) Ester; CAS Number: 26040-51-7.Available: http://ofmpub.epa.gov/oppthpv/quicksearch.display?pChem=102247 [accessed 22 April 2012].

[r60] U.S. EPA (2009b). Polybrominated Diphenyl Ethers (PBDEs) Action Plan.. http://www.epa.gov/oppt/existingchemicals/pubs/actionplans/pbdes_ap_2009_1230_final.pdf.

[r61] Van den Eede N, Dirtu AC, Ali N, Neels H, Covaci A (2012). Multi-residue method for the determination of brominated and organophosphate flame retardants in indoor dust.. Talanta.

[r62] Visser TJ, Fekkes D, Docter R, Hennemann G (1979). Kinetics of enzymic reductive deiodination of iodothyronines. Effect of pH.. Biochem J.

[r63] Ward JM, Peters JM, Perella CM, Gonzalez FJ (1998). Receptor and nonreceptor-mediated organ-specific toxicity of di(2-ethylhexyl)phthalate (DEHP) in peroxisome proliferator-activated receptor α-null mice.. Toxicol Pathol.

[r64] Watkins DJ, McClean MD, Fraser AJ, Weinberg J, Stapleton HM, Sjödin A (2011). Exposure to PBDEs in the office environment: evaluating the relationships between dust, handwipes, and serum.. Environ Health Perspect.

[r65] Wu N, Herrmann T, Paepke O, Tickner J, Hale R, Harvey LE (2007). Human exposure to PBDEs: associations of PBDE body burdens with food consumption and house dust concentrations.. Environ Sci Technol.

[r66] Yanik SC, Baker AH, Mann KK, Schlezinger JJ (2011). Organotins are potent activators of PPARγ and adipocyte differentiation in bone marrow multipotent mesenchymal stromal cells.. Toxicol Sci.

[r67] Yuan J, Chen L, Chen D, Guo H, Bi X, Ju Y (2008). Elevated serum polybrominated diphenyl ethers and thyroid-stimulating hormone associated with lymphocytic micronuclei in Chinese workers from an E-waste dismantling site.. Environ Sci Technol.

[r68] Zhou T, Ross DG, DeVito MJ, Crofton KM (2001). Effects of short-term in vivo exposure to polybrominated diphenyl ethers on thyroid hormones and hepatic enzyme activities in weanling rats.. Toxicol Sci.

